# High-Intensity Functional Training (HIFT): Definition and Research Implications for Improved Fitness

**DOI:** 10.3390/sports6030076

**Published:** 2018-08-07

**Authors:** Yuri Feito, Katie M. Heinrich, Scotty J. Butcher, Walker S. Carlos Poston

**Affiliations:** 1 Department of Exercise Science and Sport Management, Kennesaw State University, Kennesaw, GA 30144, USA; 2 Department of Kinesiology, Kansas State University, Manhattan, KS 66506, USA; kmhphd@ksu.edu; 3 School of Rehabilitation Science, University of Saskatchewan, Saskatoon, SK S7N 2Z4, Canada; scotty.butcher@usask.ca; 4 Institute of Biobehavioral Health Research, National Development and Research Institute, Leawood, KS 66211, USA; carlosposton@hopehri.com

**Keywords:** athletes, military, first responders, exercise, general physical preparedness

## Abstract

High-intensity functional training (HIFT) is an exercise modality that emphasizes functional, multi-joint movements that can be modified to any fitness level and elicit greater muscle recruitment than more traditional exercise. As a relatively new training modality, HIFT is often compared to high-intensity interval training (HIIT), yet the two are distinct. HIIT exercise is characterized by relatively short bursts of repeated vigorous activity, interspersed by periods of rest or low-intensity exercise for recovery, while HIFT utilizes constantly varied functional exercises and various activity durations that may or may not incorporate rest. Over the last decade, studies evaluating the effectiveness of HIIT programs have documented improvements in metabolic and cardiorespiratory adaptations; however, less is known about the effects of HIFT. The purpose of this manuscript is to provide a working definition of HIFT and review the available literature regarding its use to improve metabolic and cardiorespiratory adaptations in strength and conditioning programs among various populations. Additionally, we aim to create a definition that is used in future publications to evaluate more effectively the future impact of this type of training on health and fitness outcomes.

## 1. Introduction

High-intensity interval training (HIIT) refers to an exercise program that is characterized by relatively short bursts of vigorous activity, interspersed by periods of rest or low-intensity exercise for recovery. HIIT is primarily applied utilizing aerobic exercise, such as a running on a treadmill and cycling on an ergometer [1,2]. Even though this type of training has been used among athletes since the later part of the 20th century [3], it recently has gained momentum among fitness enthusiasts, and has been identified as a “Top 10 Fitness Trend” in 2018 [4]. 

Over the last decade, studies evaluating the effectiveness of HIIT programs have shown improvements in metabolic and cardiorespiratory adaptations [5,6,7,8]. Additionally, Burgomaster and colleagues [9] reported significant improvements in time to exhaustion after only six sessions of HIIT over a two-week period. This increase in endurance is most noteworthy as it was accomplished without an increase in maximal oxygen consumption (VO_2max_), which suggests “peripheral” adaptations, such as changes in carbohydrate metabolism, and enzymatic activity within the muscles might be responsible for these changes [10].

Although several investigators have substantiated the benefits of HIIT, less is known about the effects of a relatively new training modality, known as high-intensity functional training (HIFT). HIFT emphasizes functional, multi-joint movements via both aerobic and muscle-strengthening exercises [11]. HIFT can be modified to any fitness level and elicits greater muscle recruitment than repetitive aerobic exercises, thereby improving cardiovascular endurance, strength, and flexibility [11,12,13].

Most studies using the HIFT methodology have used a CrossFit training template [11,13,14], which is based on the concept of increased work capacity over time [15] while using a variety of exercise modalities including mono-structural (e.g., running, rowing, etc.), as well as body weight movements (e.g., squats, push-ups, etc.) and weightlifting derivatives (e.g., snatch, shoulder press, deadlift, etc.). Recently, several investigators have examined the effects HIFT-based programs after multiple weeks of training, and have shown significant improvements in maximal oxygen consumption (~12%) [11,12], decreases in body fat (~8%) [11,16], as well as improvements in bone mineral content (~1%) [16] after 16-weeks of HIFT. In addition investigators have reported higher levels of enjoyment [14,17], between HIFT participants and those engaged in more traditional resistance training programs, as well as a greater sense of community [18,19], which facilitates initiation and adherence to exercise training [20].

Therefore, we propose the definition of HIFT as a training style [or program] that incorporates a variety of functional movements, performed at high-intensity [relative to an individual’s ability], and designed to improve parameters of general physical fitness (e.g., cardiovascular endurance, strength, body composition, flexibility, etc.) and performance (e.g., agility, speed, power, strength, etc.). Thus, and with this definition in mind, the aims of this paper are to: (1) detail what we believe should constitute HIFT modalities; (2) provide a historical perspective of how HIFT programs have developed over the last decade; (3) provide differentiation between current high-intensity protocols; and (4) examine the application of HIFT training. 

Considering the limited research in the area, our goal is to provide a “working definition” of this style of training so that researchers around the world can equally refer to the same type of training and continue to study and expand our understanding of HIFT. Even though several reviews of the pertinent literature exist, to our knowledge, this is the first manuscript that provides a working definition of what should constitute a HIFT program. To date, investigators have used several terms to identify this type of training (e.g., high-intensity power training, multimodal high-intensity training, functional high-intensity training, etc.). Therefore, as the training modality continues to gain interest in the scientific community, a formal definition is warranted to better assess and compare the effectiveness, safety, and long-term effects of these training programs in future studies, considering the potential impact on public health outcomes.

## 2. HIIT vs. HIFT: Key Differences

A common misconception about HIFT is that it is synonymous with HIIT. While HIFT and HIIT share some conceptual commonalities, such as the high-intensity nature of the training, there are distinct differences in their methodologies that result in important differences in physiological responses and adaptations. The most important differences are in terms of the use of functional movement patterns and resistance-based exercises, as well as the prescription of rest intervals.

Typically, HIIT protocols use modalities that are unimodal in nature (i.e., running, cycling, rowing, etc.), whereas HIFT protocols are defined using multimodal and “functional” exercises. Although there are many definitions and criteria that may be used to describe functional exercises, it has been proposed that functional exercises are those that involve whole body, universal motor recruitment patterns in multiple planes of movement such as squats, deadlifts, cleans, snatches, pull-ups, vertical jumps, and more [12,14,21]. In more traditional training, these types of powerlifting, weightlifting, and gymnastics exercises are performed for prescribed sets and repetitions with a long recovery time and are assumed to not result in a significant cardiovascular response with either acute or chronic exercise [22]. However, when prescribed in a continuous circuit or interval format, and conducted at high intensity, these exercises are potent stimuli for not only improving muscle strength and power, but cardiovascular, aerobic, and anaerobic adaptations as well [22]. 

Limited research exists comparing the two training modalities; however, studies using HIFT methods specifically have resulted in significant changes in body composition [14,23], muscle strength and power [12,24,25], as well as aerobic capacity [11,12,26]. Recently, Buckley et al. [27] compared the training response of multimodal-based HIIT (similar to HIFT; MM-HIIT) with those of unimodal-based HIIT (rowing ergometer; Row-HIIT) in women. Both groups completed six weeks of training three times per week and each protocol included six-rounds of one-minute intervals completed in an “all-out” manner, with a three-minute passive cooldown between rounds. Whereas the Row-HIIT completed multiple intervals on the rowing ergometer, the MM-HIIT group completed several repetitions of a heavy strength movement (squats, deadlifts, presses, etc.), a moderate load power/accessory movement (lunges, push-ups, ring rows, push presses, etc.), and a metabolic conditioning finisher (box jumps, burpees, ball slams, battle ropes, etc.) during the 60 s work intervals. While both the MM-HIIT and Row-HIIT groups significantly improved aerobic capacity (7% vs. 5%, respectively), and anaerobic power (15% vs. 12%, respectively), after six weeks of training, only the MM-HIIT group improved muscle strength, power, and muscular endurance [27]. Therefore, it appears that multimodal-based HIIT/HIFT provides similar improvements in aerobic and anaerobic adaptations as more traditional, unimodal HIIT programs, with the added benefit of significant improvements in muscle performance that are not typically observed with either HIIT [27] or continuous [28] aerobic training.

The second major difference between HIFT protocols and more traditional HIIT protocols is the exclusion of a defined rest interval. Many HIFT workouts are based on completing a certain number of repetitions in the fastest time possible (i.e., repetitions for time) or on completing a series of exercises within a given time frame for as many repetitions as possible (i.e., As Many Repetitions As Possible; AMRAP). The nature of each HIFT workouts means that individuals will require rest or recovery breaks throughout the workout based on their current fitness levels; therefore, these breaks are often short and not prescribed, thereby taken as in “as needed” basis throughout the exercise bout. This differs from most HIIT protocols in that they often have recovery intervals at specific points in time, which are typically longer. For example, one of the more traditional HIIT protocols is the completion of multiple “all-out” 30 s sprint intervals on a cycle ergometer, repeated every 4.5 min for 4–6 rounds (repeated Wingate training) [10]. 

The differences in recovery time during various interval protocols may have important implications for inducing training-related adaptations. For example, Cochran et al. [29] performed a two-part study where, in the first part, they compared the acute responses of four repeated Wingate tests and a continuous high-intensity bout of equal work (a total of around four-minutes of all-out work on a cycle ergometer). The acute mitochondrial responses were similar between protocols, which may suggest the potential for similar adaptations. However, unlike previous studies where significant improvements in mitochondrial enzymes had been reported after chronic HIIT [30,31], Cochran et al. [29], in their second study within this paper, studied the chronic adaptations to six weeks of training using only the continuous protocol and reported that this protocol did not induce significant chronic mitochondrial adaptations that are typically observed with HIIT training. Nonetheless, while significant changes in mitochondrial proteins were not present after six weeks of training, the continuous training group showed significant increases in cycling time trial performance and maximal aerobic capacity that are similar in magnitude to previous reports of adaptations to HIIT protocols [29]. The discordance between the acute and training-related responses to these protocols presents an intriguing aspect of HIFT vs. HIIT training and their relative adaptations that requires more study.

Although several studies exist, the magnitude of the literature related to this topic is a scarce. Butcher and colleagues [32] compared a popular HIFT protocol (AMRAP of 5 pull-ups, 10 push-ups, and 15 body weight squats for 21 min) with a multimodal HIIT protocol (8 bench press, 10 pull-ups, and box jumps for the remainder of a 60 s interval with a 3-min recovery time for 6 sets) and found that while rate of perceived exertion responses were similar between the two-protocols (16 ± 1.8 vs. 18 ± 1.5, *p* > 0.05), heart rate responses were quite different. Whereas the HIFT protocol resulted in heart rates around 90% of age-predicted maximum throughout the 21-min workout, the average heart rates for the multimodal HIIT protocol were around 76% of age-predicted maximum, with only the peaks for each interval approaching 90% maximum. This suggests that a HIFT program would provide greater physiological stimulus over a 21-min workout than a HIIT protocol of comparable time. In addition, the long-term ability of a participant to maintain such high percentage of maximal heart rate would result in chronic cardiovascular adaptations, which will result in reduction of chronic disease risk factors and mortality risk [33]. These two points, however, require additional investigation within the HIFT literature.

Most recently, Sperlich et al. [34] compared the psycho-physiological responses associated with a circuit-like, multi-joint, high-intensity program (Circuit_HIIT_) compared to a low-intensity high-volume exercise program (Circuit_Combined_) among a group of inactive women during nine-weeks. Even though investigators reported improvements in body composition, strength, power, and quality of life in both groups, the Circuit_HIIT_-based protocol alone resulted in greater increases in aerobic capacity, along with greater perceptions of pain, whereas the Circuit_Combined_ increased perception of general health. Together, these studies suggest that the cardiovascular stress and potential for adaptation may be different between protocols that are continuous compared to those that are interval in nature, regardless of intensity. 

Kliszczewicz et al. [35] examined the oxidative stress of a single HIFT session including 20 min of 5-pull-ups, 10 push-ups, and 15 air-squats compared to a high-intensity treadmill session of 20 min of running at 90% maximal heart rate. Even though significant increases in oxidative markers were seen following each of the two exercise bouts, no significant differences were observed between the two modalities. Thus, the investigators concluded that when matched by time and intensity, the 20-min HIFT session, produced a physiological stress response that was similar to treadmill running. More recently, Kliszczewicz et al. [36] compared the effect of a short (<5 min) and long (15 min) HIFT workout on plasma metabolic markers. Even though significant changes in glucose, insulin, epinephrine and norepinephrine were observed immediately after each HIFT bout, no significant differences were reported between the two HIFT workouts, suggesting that perhaps even less than five-minutes of HIFT may be beneficial for those with glucose metabolism impairment [36]. Additionally, it is worth nothing that of the four markers presented, only insulin markers remained lower than pre-exercise values 1-h after the completion of the exercise bout. Additional studies should be conducted to elucidate the potential metabolic effects of this type of training modality among those with metabolic impairment.

While the evidence presented suggests differences in acute and chronic responses between traditional HIIT and HIFT protocols, the scarce data currently available comparing both training protocols prevents us from providing clear evidence to suggest one training program is superior to the other; however, it seems that HIFT protocols may allow for multiple performance and physiological adaptations that are not observed by solely training using unimodal HIIT methodology. Therefore, practitioners should consider the adaptations desired by their trainees when selecting training methods.

## 3. Historical Perspective of HIFT

Due to the physical demands of various “tactical athlete” populations (e.g., military, fire service, etc.), training needs have changed and adapted over time, with a clear “grass-roots” interest in and demand for improving fitness for job duties, and law enforcement (i.e., combat, fire suppression and rescue) [37]. For example, there has been a shift to the development and use of both combat readiness tests in the military and physical agility tests used in the fire service and law enforcement, which mimic actual job-related tasks rather than traditional fitness tests, which typically include a run, bodyweight exercises (e.g., sit-ups and push-ups), and a flexibility measure [38]. Concurrently, various specialized tactical fitness programs for the military, fire service, and law enforcement have been developed [21,37].

In line with the documented abilities required by combat personnel [39], Batchelor [40] and Lowman [41] documented the most important physical tasks involved in combat included lifting from the ground, lifting overhead, pushing, pulling and/or climbing, rotation, jumping and landing, lunging, marching, running, and changing direction. With these needs in mind, Heinrich and colleagues [12] compared the effects of a traditional military physical training program and a circuit-style HIFT program called Mission Essential Fitness (MEF) among active duty Army personnel. After eight weeks of training, the MEF group significantly increased their strength, aerobic capacity, and flexibility compared to the traditional military physical training group. 

However, military personnel, firefighters, and law enforcement officers who train to improve their operational readiness and fitness are not training solely to improve aerobic or strength parameters [42], as the physical demands of their jobs involve a broad range of fitness domains necessary to perform the mission at hand [42]. Effective fitness programming should address all these critical domains [21,37,43,44]. Recently, Haddock and colleagues [37], Poston and colleagues [21], and with Hodzovic [45] describe how programs that combine aspects of resistance training with aerobic and bodyweight exercises have excellent potential to improve both anaerobic and aerobic capacity in a more time- and volume-efficient manner than traditional military training. This introduces HIFT as valuable and appropriate training program for military personnel. 

To our knowledge, the first mention of HIFT as an intervention strategy among non-military personnel was made by Heinrich and colleagues [14]. In that study, investigators sought to compare markers of enjoyment and adherence between individuals enrolled in a HIFT program and those in a traditional moderate-intensity aerobic and weight training group. After eight weeks of training, those in the HIFT group not only spent significantly less time exercising (only 39 min per week, compared to 189), but they also had higher exercise enjoyment and intentions to continue (100%) than the traditional group (56%). These findings are significant, as they provide evidence that HIFT exercise may be more enjoyable than traditional exercise programs, which could have potential public health implications. Among military populations, such as the US Army, criticisms of the current physical training program exist, including insufficient preparation for combat and lack of training in multiple fitness domains [40]. Thus, exercise training is often geared toward passing the Army’s fitness test rather than combat preparedness [40], and this disconnect negatively impacts soldier motivation for the exercise training program.

## 4. Utilization of HIFT Programs in the General Population

The rapid growth in popularity of HIFT has public health implications, considering that less than 21% of United States adults meet current aerobic and muscle-strengthening physical activity guidelines [46]. Exercise adherence is a key area of focus because many exercise programs have high dropout rates [47]. Although previous research has addressed exercise adherence in general [48,49], there is a lack of extant information on adherence to high-intensity exercise programs that incorporate both aerobic and muscle-strengthening activities, such as HIFT. As well, limited information is available about key factors that influence initiation of high-intensity exercise in the first place [11]. It is well established that satisfaction of innate psychological needs (i.e., competence, autonomy, and relatedness) is the product of intrinsically motivated behaviors [50], whereas extrinsic factors (e.g., external rewards) can undermine intrinsic motivation [51]. Therefore, intrinsically motivated behaviors are a strong predictor of overall exercise adherence [52].

As mentioned, Heinrich and colleagues [14] were the first to use HIFT as an intervention strategy among non-military personnel. In that study, investigators sought to compare markers of enjoyment and adherence between individuals enrolled in a HIFT program and those in a traditional moderate-intensity aerobic and weight training group. After eight weeks of training, those in the HIFT group not only spent less time exercising, but they also had higher exercise enjoyment and intentions to continue than the traditional group. Moreover, Heinrich and associates [14] postulated that intrinsic motivation facilitated exercise initiation for the participants enrolled in this study. As such, individuals were more likely to be intrinsically motivated to adhere to an activity if they had feelings of high enjoyment, made social connections, and experienced low anxiety about the activity [53]. Accordingly, HIFT exercise adherence has been positively correlated with enjoyment [14,19,53], social support [18,54], and intrinsic motivation [14,19].

Overall, intrinsic motives (e.g., challenge and enjoyment) have been ranked significantly higher by HIFT participants than those completing other types of resistance exercise in a group, alone, or with a personal trainer [17]. Improving physical abilities (e.g., strength, fitness), health-related factors (e.g., prevention, being/remaining healthy), and functional skills seem to be key participation motives for HIFT participants [18,19,55]. Additionally, affiliation, challenge, and enjoyment motives have been rated significantly higher for HIFT participants than those in other types of resistance exercise groups. Conversely, appearance, health pressures, ill-health avoidance, positive health, and weight management were rated significantly lower for HIFT participants (mean age = 30.7 years) than those completing resistance exercise with a personal trainer (mean age = 47.2 years) [17].

Intrinsic regulation, content of intrinsic goals, and competence need satisfaction have positively predicted HIFT participation frequency, although participation was negatively predicted by external regulation [56]. More specifically, the basic psychological needs of autonomy, relatedness, and competence have positively predicted regulation of HIFT participation, with those who participated less often reporting lower levels of each [19,57]. However, the frequency of weekly HIFT participation has not been significantly correlated with affect, well-being, or body awareness [55]. In contrast, Bycura and colleagues [19] reported factors such as revitalization, enjoyment, affiliation, and competition to be positively correlated with length (i.e., years) and frequency (i.e., weekly) of participation in HIFT programs. They surmised that the social components of HIFT programs, including group exercise, may greatly impact overall adherence and participation [19].

Using the application of achievement goal theory within a HIFT setting, Partridge, Knapp, & Massengale [58] found that females and participants with less than six months of experience were significantly more likely to report higher mastery-based goals, while males and those with more experience were significantly more likely to report higher performance-based goals [58]. These findings suggest that the steep learning curve associated with the different components of constantly varied HIFT workouts may necessitate a focus on mastery goals for females and newer participants [58]. Awareness of participant goals is useful to help facilitate adherence as well as for linking like-minded participants together.

Fitness centers, or gyms, are social institutions that offer opportunities for like-minded individuals to interact outside of the home or work setting, which helps enhance a sense of belongingness [59]. Among HIFT programs, CrossFit training is the most common, and aims to be more than a fitness program or gym, with an emphasis on community [60]. The physical layout of HIFT facilities differ from those of traditional gyms in their value of floor space and moveable functional fitness equipment as compared to electronic cardio equipment with television screens and stationary weight machines [60,61]. Within CrossFit gyms, or “boxes” as they are typically called, equipment is minimal and interaction with others is expected and encouraged, as compared to other types of gyms and group fitness classes where people create virtual boundaries by listening to headphones or claiming a physical space for their own [60]. Indeed, some traditional group fitness classes are a “space where individuals come together to exercise alone in a group setting” [62]. When HIFT uses the group exercise model and group induction process, greater social capital and community belongingness occur, but it is possible that people more open to social interaction are attracted to HIFT in the first place [61]. CrossFit training participants establish identity through shared norms, named workouts, attire, and shared language [20,60]. Involvement in CrossFit training is thought to go beyond the gym where through this “reinventive” institution, individuals can better their lives overall–becoming the “best possible versions of themselves” [60]. 

Social capital is described as the ability to make connections with others in a social network, where trust, reciprocity and cooperation are paramount [63]. In this regard, researchers studying the contextual and social environment of HIFT compared to traditional gyms in the UK found that social capital and sense of community were significantly higher among those who participated in HIFT (e.g., CrossFit training) than those who attended traditional gyms [61]. Moreover, social capital and community belongingness were both positively correlated with weekly gym attendance for each gym type, suggesting that an individual’s ability to make connections and build trust and cooperation with other participants were important factors for maintaining exercise adherence [61]. However, when trying to determine what factors were most influential in predicting gym attendance, regression analysis showed that social capital, community belongingness and gym type only accounted for 6% of the variance, suggesting that gym attendance was influenced by many factors other than those examined by the authors [61]. As described by the authors, those in the HIFT group were younger and reported fewer years of membership compared to the traditional gym group, which may be important, considering it is well established that younger individuals are more proactive in building social networks compared to their older counterparts [64]. 

Perhaps the nature of HIFT environments provide unique opportunities for participants to build meaningful relationships and social networks (e.g., working out in groups, tracking and sharing workout results, social activities outside the gym, etc.). Recent evidence from Heinrich and colleagues [20] examined key factors that may affect participation in HIFT from the viewpoint of coaches. Findings suggest that key elements, such as environmental and social factors, along with physical and psychological changes experienced by the participants are thought to influence and promote exercise adherence [20]. Moreover, a focus on community building, reducing intimidation and cost, goal setting, monitoring progress, and social support were key factors for facilitating participation [20].

As a form of HIFT, CrossFit training is considered both fitness training and a sport, which allows for the public measurement and recording of workouts [65]. Performing workouts facilitates “peak experiences of flow”, especially when performed in supportive group settings [65]. Participants who engage in CrossFit training view the body as a machine through the immersive model of sport (i.e., positive effects from competition with in-group bonding empowering disenfranchised people/groups where gender is on a continuum), which is a healthful practice [65]. Recently, researchers examined if individuals engaged in CrossFit training might have higher than usual levels of exercise addiction–or obsessive need to complete increasing amounts of exercise despite pain or injury, resulting in negative consequences–which ranges between 3–29% in different types of sports [66]. The authors concluded that among the 603 participants (55% male) engaged in CrossFit training, younger (<age 30) men with higher exercise training volumes were more likely to experience exercise addiction; however, the overall prevalence of self-reported exercise addiction was 5%, which was similar to other sports [66]. Those with higher exercise addiction scores were more likely to “exercise despite pain/injury,” feel “guilt when missing exercise,” have “obsessive exercise,” and “take medication to exercise” [56]. Interestingly, greater “obsessive exercise” was positively associated with having “conflicts with family.” The 5% rate found in the study was slightly lower than previous rates for football [soccer] players (7.1%) and individual fitness participants (9.7%) ages 16–39 [66].

Another appeal of HIFT also is based on the idea of empowering women in a postfeminist context, including women defeating men in workouts as well as showing strong women accomplishing difficult tasks [67]. Despite its focus on functional fitness, CrossFit marketing has seemed to focus more on attractive bodies than functional ones–which was in accord with the obsession on femininity in the postfeminist media culture [67]. It is expensive to participate in CrossFit training at an affiliate and composite media from CrossFit promotes a woman who is “not too old, already or formerly very active, overwhelmingly White, and have access to the resources needed to be successful, especially money, time, and energy” [67]. However, more recent images have been more inclusive of multiple body types, ages, and health conditions (www.crossfit.com). 

HIFT programming is typically associated with younger healthy individuals, considering the high-intensity nature of the training. In a recent study, Feito and colleagues [68] examine changes in body composition, bone metabolism, strength, and skill-specific performance over 16- weeks of HIFT among a group of apparently healthy adults. Overall, improvements of body fat percent (−6.5 ± 14.2 %fat) and bone mineral content among women (+0.7 ± 1.9 g/cm^2^) were observed. The latter finding, although preliminary, considering the cross-sectional nature of the study, might suggest this training modality could have potential osteogenic effects in a relatively faster time period than traditional exercise programs [68]. Future studies should consider this training modality among populations with impaired bone metabolism (e.g., osteopenia, osteoporosis, etc.).

To our knowledge, Heinrich et al. [11] were the first to report the potential benefits of HIFT among a clinical population. In that study, researcher sought to determine how cancer survivors may benefit from as little as five-weeks of HIFT training. Participants reported significant improvements in emotional functioning, as well as improvements in measured body composition (increased lean mass, decreased fat mass and body fat percentage) and five of seven functional performance tests [11]. Additionally, cancer survivors who adhered to the program found the HIFT group exercise program to be acceptable and appealing [11]. Moreover, even though the program was well received by these participants, investigators noted the importance of properly screening and individualizing exercise programs, which allowed for modifications for cancer survivors participating in HIFT and required education and appropriate training for coaches [11].

More recently, Nieuwoudt et al. [69] and Fealy et al. [70] provided insight to the effect of 6-weeks of HIFT on beta-cell function [69] and insulin resistance [70] among a group of overweight/obese individuals with type 2 diabetes mellitus. Investigators reported significant improvements in beta-cell function and insulin processing [69], and insulin sensitivity [70]. In addition, and similar to other studies with healthy populations, Nieuwoudt and colleagues also reported significant improvements in oxygen capacity (2.43 ± 0.12 vs. 2.81 ± 0.15 L/min pre and post, respectively) and physical performance (223 ± 12 vs. 282 ± 11 repetitions pre and post, respectively) after 6-weeks of training. Perhaps more importantly from a public health perspective, is Fealy et al. significant decrease in metabolic syndrome z-score (6.4 ± 4.5 vs. −0.2 ± 5.2 AU; *p* < 0.001), suggesting that participants overall significantly improved their overall risk of developing cardiovascular disease and mortality [71]. Future studies should consider these findings and design-controlled interventions that can elaborate on these findings and evaluate the public health impact of this type of interventions among individuals with multiple risk factors for cardiovascular disease and metabolic syndrome.

The level of experience and training of HIFT coaches was recently investigated by Waryasz et al. [72] and Maxwell and colleagues [73]. A survey evaluating CrossFit instructor credentials and exercise programming practices found significant variability in training methods and education/certifications of 193 instructors [72]. Even though the vast majority of coaches interviewed had a minimum of a bachelor’s degree and had completed a CrossFit Level 1 certification course [72,73], 26% of those interviewed by Maxwell had an associate’s degree or less and 48.8% of those in Waryasz’s study did not have another personal training certification of any type. In addition, large variations were observed in the level of experience reported by Waryasz and colleagues [72], as the mean years of experience was 8.5 years, with a standard deviation of 8.5 years, which suggest some of those interviewed were recently starting in the field. As such, it has been suggested that some coaches at HIFT facilities may not have the requisite exercise science degree, training, or experience to adequately protect their members from injury risks, particularly those from high repetition high-stress movements [74]. Exercise modifications may not always be used when needed due to participant ego or lack of coaching knowledge, and high risk/low benefit exercises (e.g., kipping toes-to-bar, single-leg squats) are included in some workouts [74]. 

Many benchmark CrossFit workouts contradict physiological training principles and scientific recommendations for developing/enhancing physical fitness via muscular strength and power or plyometric training [74]. Additionally, Maxwell et al. [73] reported on the level of sport nutrition knowledge among 289 HIFT trainers and found they had only moderate sport nutrition knowledge, with trainers answering about 65% of questions correctly. Those who answered correctly, were most knowledgeable about energy needs/recovery (75% correct) and micronutrients (70% correct), but less knowledgeable about hydration (55% correct) and macronutrients (50% correct). However, this is not surprising given that the last two components are significantly disputed by CrossFit, Inc. in opposition to current nutritional and hydration guidelines [75,76].

Aside from being utilized in health and fitness settings, HIFT also has been utilized in education facilities. An evaluation of college credit courses for weight training and HIFT found that muscular power, via standing long jump, increased significantly more for traditional weight training students, while HIFT students increased muscular power significantly more than students who completed weight training on their own [24]. Traditional weight training students also increased upper body power in the YMCA bench press test over those in the HIFT groups and those doing weight training on their own [24]. However, it is worth pointing out that the HIFT group had the greatest improvement in lower body endurance, as performed in the 1-min squat test. Sibley [77] provided a template for utilizing HIFT for sport education in a middle or high school in order to “challenge and motivate students to improve their fitness levels” (p. 48), in order to make fitness approachable, realistically challenging, and fun.

## 5. Application of HIFT with Tactical Athletes

Due to the physical demands of various “tactical athlete” populations (e.g., military, fire service, etc.), training needs have changed and adapted over time, with a clear “grass-roots” interest in and demand for improving fitness for job duties, and law enforcement (i.e., combat, fire suppression and rescue) [37]. For example, there has been a shift to the development and use of both combat readiness tests in the military and physical agility tests used in the fire service and law enforcement, which mimic actual job-related tasks rather than traditional fitness tests, which typically include a run, bodyweight exercises (e.g., sit-ups and push-ups), and a flexibility measure [38]. Concurrently, various specialized tactical fitness programs for the military, fire service, and law enforcement have been developed [21,37].

In line with the documented abilities required by combat personnel [39], Batchelor [40] and Lowman [41] documented the most important physical tasks involved in combat included lifting from the ground, lifting overhead, pushing, pulling and/or climbing, rotation, jumping and landing, lunging, marching, running, and changing direction. With these needs in mind, Heinrich and colleagues [12] compared the effects of a traditional military physical training program [Army Physical Fitness Readiness Training (APRT)] and a circuit-style HIFT program [MEF] among active duty Army personnel. Briefly, the APRT is typically carried out 5-days-per-week, and includes exercisses focusing in mobility, strenght, and endurance, including a warm-up, 50-min of exercise including aerobic and resistance training, and a cooldown [78,79]. The MEF program includes a total of 15 different exercises composed of multiple multimodal movements focusing on strength, power, speed, and agility [12], including Olympic lifts, squats, bench press, and pull-ups, plyometrics, lower body movements (e.g., weighted walking lunges), upper body movements (e.g., band bicep curls), and core exercises (e.g., plank with feet elevated on a medicine ball) perfomed in a circuit-like format of 60–90 s each per station, with little or no rest between stations for 45 min. After eight weeks of training, the MEF group significantly increased their bench press strength (13.2 ± 12.1 versus 2.7 ± 11.5 pounds; *p* = 0.001), aerobic capacity resulting in improved 2-mile run time (−89.91 ± 70.23 versus −15.33 ± 69.16 s; *p* = 0.003), and flexibility (0.6 ± 1.3 versus −0.5 ± 1.5 inches; *p* = 0.003) compared to the traditional APRT group. 

However, military personnel, firefighters, and law enforcement officers who train to improve their operational readiness and fitness are not training solely to improve aerobic or strength parameters [42], as the physical demands of their jobs involve a broad range of fitness domains necessary to perform the mission at hand [42]. Effective fitness programming should address all of these critical domains [21,37,43,44]. Recently, in their respective reviews, Haddock et al. [37], and Poston and colleagues [21] described how programs that combine aspects of resistance training with aerobic and bodyweight exercises have excellent potential to improve both anaerobic and aerobic capacity in a more time- and volume-efficient manner than traditional military training. This introduces HIFT as valuable and appropriate training program for military personnel. 

Concerns have been raised about the relevance and efficacy of traditional fitness approaches to improve fitness and produce operational readiness among many military, fire service, and law enforcement professionals [21,37]. As noted by Haddock et al. [37] and Poston et al. [21] in their respective reviews, there are four specific concerns with traditional fitness programs for these populations. First, it seems that traditional fitness programs are heavily focused on improving aerobic conditioning by emphasizing running medium to long distances, especially for the military. Second, it does not appear that traditional fitness training and/or training programs that emphasize aerobic conditioning provide the fitness capacity relevant for the full spectrum of physical demands of combat, fire suppression and rescue, and law enforcement tasks. Third, traditional fitness training approaches do not take into account the highly variable and unpredictable physical demands required of tactical athletes when engaged in operational situations (i.e., running on a treadmill for an hour does not mimic going into a structure fire while wearing 75 lbs of protective equipment, including a face mask and oxygen tank; nor does it mimic rucking with a 75 lb field pack in a deployed environment or having to rescue carry an injured combatant); and fourth, traditional fitness programming, particularly programs that emphasize high running volumes, result in troubling and high rates of injury [21,37,43,80], which not only impact the individual, but can also be costly to the entire team.

Haddock and colleagues [37] recently reviewed the application of HIFT approaches to military personnel and the outcomes associated with programs tested by the Canadian Infantry (Combat Fitness Program which was based on CrossFit), the US Army Command and General Staff College (CrossFit), the Naval Health Research Center (Combat Conditioning Trial Program), the United States Air Force (USAF) Combat Controller training program, and the Mission Essential Fitness program tested in US Army troops. The potential benefits of these type of HIFT programs, as identified by the authors, included reduced training volumes and subsequent injury risk, improved fitness across multiple domains (e.g., strength, power, agility, flexibility, both aerobic and anaerobic capacity, and muscle endurance), potential for improved adherence due to the variability in programming, use of more efficient full-body functional movements that may better prepare tactical professionals for real world applications, which could be termed improved general physical preparedness, and reduced body fat/improved body composition [37]. 

Additionally, a few years earlier, O’Hara and colleagues [81] reviewed studies of different HIFT approaches that they termed “Non-traditional Training Modalities”, including CrossFit, Kettlebell Training, lower heavy extremity strength training, and agility training with a focus on their potential to improve physical performance among US Air Force personnel. They concluded that programs that included heavy leg strength training and agility training improved aerobic fitness and fitness test scores (e.g., among Air Force personnel, especially among those having trouble meeting US Air Force fitness requirements); however, further research on these non-traditional training modalities was warranted [81].

Considering the evidence-based benefits for HIFT programs among military personnel, there is clearly no reason to believe that the benefits would not be applicable to other tactical occupational groups that also have significant physical demands, such as firefighters and law enforcement officers. Several studies have documented the substantial physical and metabolic demands associated with both firefighting and law enforcement and the fact that multiple fitness domains are important for successful performance of job tasks [82,83,84,85,86,87,88,89]. Rhea and associates [88] demonstrated tests of strength (both upper and lower body and grip), muscular endurance, and anaerobic capacity were tests most strongly associated with simulated firefighting tasks (e.g., hose pull, dummy drag, stair climb, etc.), while aerobic capacity was not a statistically significant correlate with any of the firefighting tasks. Similarly, Michaelides and associates [86,87] reported that muscular endurance, strength, and flexibility were the best predictors of firefighter job task performance. Beck and colleagues [84] also found that upper body strength and endurance, lower body power, torso endurance, and agility were significantly correlated with performance of law enforcement job tasks (e.g., stair ascent and descent, 159 m run, simulated rescue/arrest, etc.), although they also found aerobic capacity to be a significant correlate.

Over the last decade, several investigators have recommended that tactical professionals engage in regular fitness training to improve overall health and job performance in order to reducing injury risks and prevent deaths related to poor fitness [90,91,92,93,94,95]. Recommended training typically focuses on multiple fitness domains (e.g., aerobic and anaerobic capacity, strength, power, agility, flexibility, etc.) in recognition of the variety of tasks in which tactical professionals may have to engage (e.g., sprinting after a perpetrator, breaching a door, rescuing and carrying a person to safety, carrying a charged fire hose, etc.). However, intertwining traditional modalities while incorporating functional movements that mimic tactical job tasks and better mimic real world tactical situations, may require performance of activities impacting several fitness domains at the same time [91,92,95,96]. As noted previously, this notion of bringing together these separate training modalities into a combined training approach is central to the definition of HIFT approaches [21,45]. It is clear that HIFT approaches are applicable to the fitness needs of law enforcement officers and firefighters, similar to those studied for military members, and it is likely they would confer health and job performance benefits. Nonetheless, there is a dearth of well-designed, randomized, longer duration (i.e., four months or longer) general fitness intervention studies, particularly those focused on HIFT, among these tactical occupational groups.

### 5.1. Firefighter Fitness Studies

Abel and colleagues [97] evaluated the immediate effects of a circuit training workout on aerobic and anaerobic metabolic markers in a sample of 20 career firefighters. They also compared the results of the circuit training workout on the markers with the same outcomes among firefighters who engaged in simulated firefighting and rescue tasks. They reported that post-workout blood lactate levels were similar to those previously measured among firefighters engaging in simulated job tasks, thus concluding that the circuit workout produced a similar anaerobic response to firefighting tasks [92].

Additionally, Roberts et al. [98] and Peterson et al. [99] evaluated the effect of several weeks of training among firefighters. Roberts et al. [98] conducted a 16-week single group, pre-test, post-test training program that included periodized resistance, aerobic, and functional training with 115 firefighter recruits three days per week for one hour sessions, resulting in significant improvements in aerobic capacity (VO_2max_), upper body muscle endurance (push-ups), and flexibility. Peterson et al. [99] conducted a 9-week study comparing two fitness interventions, one using traditionally periodized resistance training and the other using an undulating/non-linear approach to programming resistance training among 14 firefighters. Both groups trained three days/week for 1–1.5 h per session. The traditional periodized program resulted in significant improvements in upper and lower body strength and power output, vertical jump height, and performance on tests simulating six firefighting and rescue tasks for both programs; however, the undulating program produced greater improvements in the overall score of the firefighting task performance test [99]. 

More recently, Pawlak et al. [94] evaluated the impact of two fitness interventions over 12 weeks in a sample of 20 career firefighters. Firefighters were assigned to either an unguided control group (no-treatment control) or a supervised and structured (2–3 day/week) scaled circuit training program that included aerobic, strength, and functional training. At the end of the study, firefighters in the supervised circuit training program had a significantly greater proportion who completed simulated firefighting tests. In addition, they experienced significantly greater improvements in body composition. 

Finally, Griffin and colleagues [100] examined the benefits of a three days per week functional training fitness program for fire service recruits during the academy and probationary periods on injury reduction in a cohort study of 109 total firefighter recruits. Injuries were ascertained from injury surveillance reports and workers compensation claims for firefighter recruits in years prior to the intervention implementation (2007–2009) and for the year the program was in place (2012). Recruits exposed to the functional fitness training program experienced significantly lower injury rates (11% vs. 54%; *p* = 0.009) and filed fewer workers compensation claims (13 claims totaling US $6679 vs. 55 claims totaling US $95,582; *p* = 0.028) than recruits who were not exposed to the program.

### 5.2. Law Enforcement Officer Fitness Studies

Similar to those studies with firefighters, several investigators have examined the role of less traditional training programs with law enforcement personnel. Crawley and colleagues [101] evaluated a 16-week multicomponent fitness intervention that included aerobic, plyometric, body weight, and resistance training three days per week in a single group of 68 police academy recruits. At the end of the 16-week program, participants demonstrated significant improvements in agility, core endurance (sit-ups), upper body endurance (push-ups), lower body peak power, and aerobic capacity (800m shuttle run test) when compared to baseline testing. 

Additionally, Cocke and associates [102] compared a traditional periodized fitness conditioning program with one that randomly assigned workouts, incorporating both strength and endurance exercises that were spontaneously selected the day of the workout, for 61 male police academy cadets. Workouts were five days per week for approximately 60 min per session over a six-month period. Although it was not clear how the cadets were randomly assigned to each condition, the investigators reported that compared to those in the traditional periodized training program, those in the randomly selected training group had significantly greater improvements on the vertical jump test, peak power output estimated by the vertical jump, and anaerobic capacity, as measured by 300m sprint time [102]. With respect to within-group changes, those in the randomized fitness program experienced significant changes over time in all 10 fitness test domains, including improvements in body composition, while the traditional training group only experienced significant changes over time in three tests (push-ups, sit-ups, and 300 m sprint time) [102].

Given that HIFT focuses on producing fitness across a broad range of domains, incorporates training with movements and apparatus typically used by tactical athletes in the field, and can have lower training volumes that traditional fitness approaches, it has been suggested that HIFT programming may be more suitable for tactical athletes who wish to develop fitness across a variety of domains that are critical to meeting the physical demands of their unique occupations [21]. In other words, while being “fit” in aerobic, muscle endurance, and flexibility domains, as typically viewed by traditional fitness programming, is important for overall health, wellness, and longevity of tactical athletes to also receive training in additional fitness domains as those provided by HIFT, to be successful. Their fitness levels must provide operational readiness and meet the rigorous physical demands of their jobs, which are substantially more than most individuals in the general public. Thus, it is suggested that fitness approaches that may be adequate for the general public are not substantive enough for tactical athletes [37].

## 6. Safety

Although it is important to point out that HIFT programing is not injury free, recent studies have provided insight into the prevalence and incidence of injury associated with these training programs. Overall, injury rates are similar, and often even lower, than those typically reported for running or other fitness activities associated with both military physical training or fitness programming commonly endorsed for use by tactical athletes. For example, Poston et al. [21] examined the available literature reporting injury incidence rates (number of injuries per 1000 h of training) and found the range in injuries related to HIFT programs were between 0.0–3.9 injuries/1000 h of training, which was lower than those reported for running (10.0 injuries/1000 h of running) [103] and several other activities such as soccer (7.8 injuries/1000 h of training) [104]. Moreover, these rates of injury were lower than the average incidence of injury among Zumba^®^ instructors (5.7 injuries/1000 h) as recently reported by Domene and colleagues [105].

Most recently, amid some controversy [106,107] several new investigations have examined the incidence of injury among individuals engaging in HIFT-based programming, such as CrossFit training. Although methods varied across studies, e.g., one was a 12-week prospective study (2.1 injuries/1000 h of training; [108]) and two were retrospective studies where participants reported injuries that occurred in the previous six months (2.3 and 1.9 injuries/1000 h of training; [109,110], injury incidence rates were consistent with those previously published and summarized by Poston et al. [21]. These findings have led other independent reviewers to conclude that the risk of injury for HIFT participants, especially those using CrossFit training as the main methodology, were comparable or lower than rates for other recreational or physical training approaches [111,112]. In fact, it was noted by Jones and colleague [80] that the best methods for reducing injury risk among military personnel were to reduce overall running volumes, substitute cross-training activities for running, and conduct running groups scaled to individual ability. 

## 7. Conclusions

Traditional exercise training programs have helped improve major components of health-related fitness (i.e., aerobic capacity, body composition, strength, etc.). However, considering the number of individuals currently not meeting the physical activity guidelines, it seems that these programs have a lack of appeal, and may not completely address fitness needs. Over the last few years, HIIT has gained significant momentum as a new training modality that seems to provide similar benefits than more traditional training programs, with a lower time commitment. This reduction of time, along with significant improvement in overall fitness, have resulted in HIIT gaining popularity over the last five-years.

More recently, a new method to achieve fitness has emerged that encompasses the intensity of HIIT programs, while incorporating functional movements that are easily reproducible, often with very little equipment. Although this type of training has been in practice for several years, limited research exists to demonstrate its effectiveness, amid its popularity. As a result, and with a goal to provide a clear definition of what should be considered a HIFT program, we have provided a working definition based on what other investigators have described in their studies—a *training style [or program] that incorporates functional, multimodal movements, performed at relatively high intensity, and designed to improve parameters of general physical fitness and performance.*

To better understand this training style, we have provided a historical perspective of HIFT programs over time, and a rational regarding why they were originally designed, and how they are different than more traditional HIIT programs, even though they share the high-intensity component. Moreover, we included their application among those in the general population, as well as among tactical athletes. As such, and in contrast with several other investigators who have used the term “extreme conditioning programs” to describe this type of training modality [113,114,115], we believe the use of the term we propose herein—high-intensity functional training—is most appropriate and should be considered for future studies investigating this style of training. 

Considering the growing focus on functional exercises, as well as the public’s interest in these type of training programs, it is important for investigators to examine and compare the effectiveness, safety, and long-term effects of these programs, as they have potential for significant public health impact. At this time, and based on current available evidence, several gaps in the literature exist. For example, further studies are needed that (1) compare HIIT and HIFT protocols (cross-sectionally and longitudinally) to determine differences between the two training modalities; (2) examine physiological responses to different HIFT workouts, as well as potential sex and age-group differences that may results from these training programs; (3) include populations with chronic disease (e.g., obesity, diabetes, heart disease, etc.), which may significantly benefit from some of the observed physiological changes reported in the literature; (4) examine the ability of HIFT programs for combat readiness preparation, compared to traditional military training programs; as well as (5) assess differences between individuals who initiate and continue HIFT participation and those who do not.

Even though the appeal of this training modality has increased over the last decade, many questions still remain unanswered and require further study. It is our hope that the evidence provided herein grants other researchers valuable insight into these programs, and our definition is used in future publications to evaluate more effectively the true reach and impact of this type of training over the years to come.
